# *Xenopus *Dab2 is required for embryonic angiogenesis

**DOI:** 10.1186/1471-213X-6-63

**Published:** 2006-12-19

**Authors:** Seong-Moon Cheong, Sun-Cheol Choi, Jin-Kwan Han

**Affiliations:** 1 Division of Molecular and Life Sciences, Pohang University of Science and Technology, San 31, Hyoja Dong, Pohang, Kyungbuk, 790-784, Republic of Korea; 2 Present address : Brookdale Department of Molecular, Cell and Developmental Biology, Mount Sinai School of Medicine, One Gustave L. Levy Place, New York, NY 10029, USA

## Abstract

**Background:**

The molecular mechanisms governing the formation of the embryonic vascular system remain poorly understood. Here, we show that Disabled-2 (Dab2), a cytosolic adaptor protein, has a pivotal role in the blood vessel formation in *Xenopus *early embryogenesis.

**Results:**

*Xenopus Disabled-2 *(*XDab2*) is spatially localized to the blood vessels including the intersomitic veins (ISV) in early embryos. Both antisense morpholino oligonucleotide (MO)-mediated knockdown and overexpression of *XDab2 *inhibit the formation of ISV, which arise from angiogenesis. In addition, we found that activin-like signaling is essential for this angiogenic event. Functional assays in *Xenopus *animal caps reveal that activin-like signals induce *VEGF *expression and this induction can be inhibited by XDab2 depletion. However, *XDab2 *MO has no effects on the induction of other target genes by activin-like signals. Furthermore, we show that the disruption of the sprouting ISV in XDab2-depleted embryos can be rescued by coexpression of *VEGF*.

**Conclusion:**

Taking together, we suggest that XDab2 regulates the embryonic angiogenesis by mediating the *VEGF *induction by activin-like signaling in *Xenopus *early development.

## Background

During embryogenesis, the formation of blood vessels is accomplished by two distinct processes called vasculogenesis and angiogenesis. In the former process, angioblasts derived from the lateral plate mesoderm migrate and differentiate into endothelial cells, thereby forming endothelial tube and primary vessels. And then the latter process involves the sprouting of new vessels from these primary vessels and their proper remodeling [1-3].

VEGF (vascular endothelial growth factor) is well known to have critical roles in the formation of blood vessels [4,5]. It affects not only the differentiation of angioblasts and the formation of endothelial tube during vasculogenesis but also the degradation of extracellular matrix and the proliferation and migration of endothelial cells during angiogenesis [6-12]. Some evidence shows that HIF-1 (hypoxia-inducible factor 1), a critical factor expressed in hypoxic condition, is involved in the induction of *VEGF *[13-17]. In addition, recent studies using some cultured cells have showed that TGFβ could induce *VEGF *gene [18-23]. However, little is known regarding the molecular mechanism by which *VEGF *induction is regulated in vivo, despite its pivotal effects on blood vessel formation.

During *Xenopus *development, flk-1, a VEGF receptor, is expressed in endothelial precursor cells which will form the major blood vessels including the posterior cardinal veins (PCV), the dorsal aorta (DA) and the vitelline veins, and *VEGF *is localized in tissues, such as hypochord 3 and somites, adjacent to the *flk-1 *expressing endothelial precursors [24]. After establishment of the primary vasculatures, *flk-1 *expression is also observed in the intersomitic veins (ISV) formed by angiogenesis. These results suggest a role for VEGF/flk-1 signaling in both vasculogenesis and angiogenesis in *Xenopus *early embryos. Supporting this possibility, ectopic expression of *VEGF *by injection of either plasmid DNA or synthetic mRNA altered the architecture of developing vasculature [24]. In addition, ectopic VEGF could act as a chemoattractant for angioblasts, suggesting that localized sources of VEGF play a role in patterning the embryonic vessels [25].

Disabled (Dab) was first known as a factor to affect the neuronal development of *Drosophila *[26]. And then Disabled-2 (Dab2), one of its mammalian isoforms, which contains an N-terminal PTB domain and a C-terminal PRD [27], was identified as a cytosolic adaptor regulating endocytosis [28]. *Dab2*-null mice reveal that it has significant roles in endodermal cell positioning and structure formation of the extra-embryonic visceral endoderm during early embryogenesis, and in adult kidney function [29,30]. It was also suggested that Dab2 could be a tumor suppressor since its expression level, which is maintained in normal ovarian cells, is remarkably decreased in ovarian carcinoma cells [31]. Moreover, Dab2 acts as an adaptor to link TGFβ receptor to Smad2 or Smad3 resulting in the promotion of TGFβ signaling pathway [32], whereas it regulates negatively the canonical Wnt/β-catenin signaling pathway [33].

In our initial attempt to address the function of Dab2 in *Xenopus *early development, we unexpectedly found that its expression is specifically restricted to blood vessels. Inhibition of Dab2 function or activin-like signaling in *Xenopus *early embryos disrupted the intersomitic veins arising from angiogenesis. Interestingly, its knockdown specifically inhibited the induction of *VEGF *gene with no effects on that of other target genes by activin-like signals. We also found that defects in intersomitic veins caused by Dab2 depletion could be rescued by coexpression of *VEGF*. Therefore, we suggest in this study that Dab2 plays pivotal roles in embryonic angiogenesis by acting as a mediator of activin-like signaling pathway for *VEGF *induction.

## Results

### Xenopus Dab2 is expressed in embryonic vasculature

**Figure 1:**
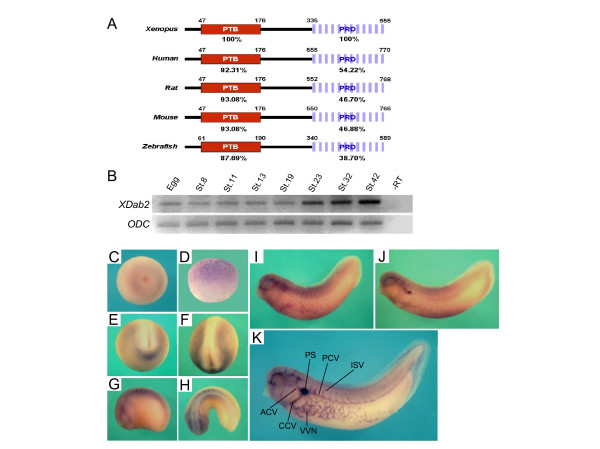
**Comparison of Dab2 homologue sequences and spatiotemporal expression pattern of *Xenopus Dab2***. (A) Alignment of *Xenopus*, human, rat, mouse and zebrafish *Dab2 *sequences. They contain the conserved N-terminal PTB and C-terminal PRD domains. The identities between the domains are shown as percentages. (B-K) The spatial and temporal expression patterns of *XDab2*. (B) RT-PCR analysis showing the temporal expression pattern of *XDab2 *in *Xenopus *early development. Stages are indicated above the lanes. ODC serves as a loading control. (C) Animal view of a one-cell stage embryo. (D) Lateral view of a cleavage stage embryo. (E) Anterior view of a neurula stage embryo with dorsal at top. (F) A neurulae viewed dorsally with anterior at bottom. (G) Lateral view of a tailbud stage embryo showing *XDab2 *expressed in presomitic region. (H) Dorsal view of a tailbud stage embryo with anterior at left. (I and J) Lateral view of late tailbud stage embryos. (K) A tadpole stage embryo in which *XDab2 *is expressed in the pronephric sinus (PS), vascular vitelline networks (VVN), anterior cardinal veins (ACV), common cardinal veins (CCV), posterior cardinal veins (PCV) and intersomitic veins (ISV).

To investigate the function of Dab2 in *Xenopus *early embryos, we first cloned a *Xenopus *orthologue of Disabled-2 (Dab2) by using PCR-based method and sequence information in EST database. *Xenopus Disabled-2 *(*XDab2*) cDNA consists of 1668 nucleotides which encode a protein of 555 amino acids (GenBank accession no. DQ367065, Fig. 1A). The predicted amino acid sequence of our clone is more similar to Dab2 orthologues of other vertebrates than Dab1 (Tab.1). Like other orthologues, it contains both N-terminal phosphotyrosine binding (PTB) domain (or phosphotyrosine interacting domain; PID) and C-terminal proline-rich domain (PRD). PTB and PRD domains show over 87% and 40% identity, respectively, across species. Furthermore, sequence alignment reveals that *XDab2 *is most similar to the short splicing isoforms (mouse, p67; rat, p59) of mammalian *Dab2 *(Table 1), which lack the motifs required for Dab2 to act as an adaptor for endocytosis in vivo.

**Table 1:** Homology comparison among Dab2 and Dabl from different species

Identity Percentage												
		Dab2							Dab1			
		
		Xenopus	Zebrafish	Mouse p96	Mouse p67	Ratp82	Rat p59	Human	Zebrafish	Mouse	Rat	Human

Dab2	Xenopus	100	47.6	41.6	57.6	41.4	57.3	44.5	35	35.5	35.5	35.9
	Zebrafish	-	100	33.5	45.2	33.9	45.5	34.2	33.3	33.4	33.3	32.9
	Mouse p96	-	-	100	71.5	94.4	67.4	82.8	27.5	31.2	31.3	31.6
	Mouse p67	-	-	-	100	67.4	94.2	57.6	32. 6	37	37.6	37
	Ratp82	-	-	-	-	100	71.6	82.7	26.9	30.8	31.4	31.1
	Ratp59	-	-	-	-	-	100	58.1	32.5	36.9	37.4	36
	Human	-	-	-	-	-	-	100	28.9	31.5	32.2	32.1

Dab1	Zebrafish	-	-	-	-	-	-	-	100	62.9	63.4	63.9
	Mouse	-	-	-	-	-	-	-	-	100	98.9	96.6
	Rat	-	-	-	-	-	-	-	-	-	100	96.8
	Human	-	-	-	-	-	-	-	-	-	-	100

XDab2 amino acid sequence is more homologous to other vertebrate Dab2 than to Dab1 and is most similar to short splicing isoforms of mammalian Dab2 (mouse p67, 57.6% identity; rat p59, 57.3% identity) as shown here.

We next examined the spatial and temporal expression patterns of *XDab2 *(Fig. 1B–G). RT-PCR analysis showed both its maternal and zygotic transcriptions throughout early development (Fig. 1B). Particularly, it increased gradually after the late neurula stages. Spatially, it was weakly expressed in the animal hemisphere at the cleavage stages (Fig. 1C and 1D) and around the anterior border of neural plate and somitic region at the neurula (Fig. 1E and 1F) and tailbud stages (Fig. 1G and 1H). At the late tailbud stages, *XDab2 *is found in vitelline vein networks (Fig. 1I). As development proceeds, it appeared in the pronephric sinus and posterior cardinal veins (Fig. 1J) and concomitantly disappeared in the somites. During the tadpole stages, its specific and strong transcriptions were observed in the vasculatures including vascular vitelline vein networks (VVN), anterior cardinal veins (ACV), common cardinal veins (CCV), pronephric sinus (PS), posterior cardinal veins (PCV) and intersomitic veins (ISV) (Fig. 1K).

### Overexpression of XDab2 affects the formation of intersomitic veins

**Figure 2:**
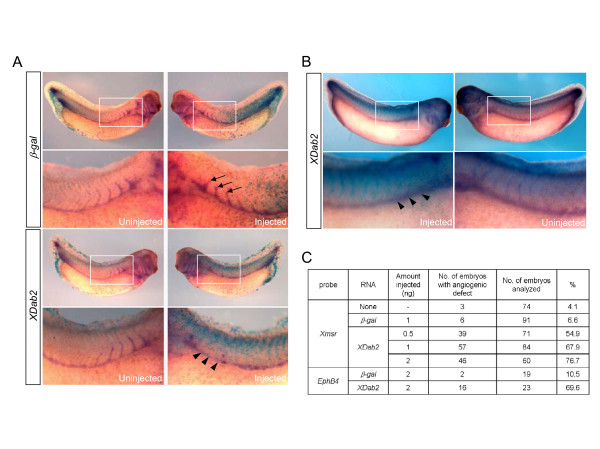
**Overexpression of *XDab2 *disrupts the sprouting of ISV**. (A and B) Injection of *XDab2 *RNA inhibits the formation of the sprouting ISV on the injected side of the embryo, with that on the uninjected side being normal. The same amount of control nuclear β-galactosidase (β-gal) RNA shows no effects on the ISV sprouts. One blastomere of two-cell stage embryos was injected with β-gal RNA as a lineage tracer with or without *XDab2 *RNA. Embryos were fixed at stage 34, stained for β-gal and then hybridized against *Xmsr *(A) or *EphB4 *(B). Arrows and arrowheads indicate the normal and disrupted ISV on the injected side of the embryo, respectively. Rectangular areas in the upper panels are enlarged in the lower panels. (C) The table showing the results from the gain-of-function analysis of *XDab2*. Overexpression of *XDab2 *causes the angiogenic defects in a dose-dependent manner.

Based on its spatial localization to vascular structures, we focused on the function of XDab2 in the formation of blood vessels during *Xenopus *early development. Thus, we first examined the effects of gain-of-XDab2 function on the formation of vascular structures in early embryos. To this end, we overexpressed *XDab2 *mRNA in one blastomere of 2-cell stage embryos and then observed its effects on vascular formation by hybridizing against *Xmsr *or *EphB4*, endothelial specific markers of *Xenopus *embryo [34,35]. As shown in Fig. 2, the formation of intersomitic veins (ISV) was disrupted on the injected side of *XDab2*-overexpressed embryos (Fig. 2A and 2B, arrowheads) in a dose-dependent manner (Fig. 2C). However, injection of β-gal RNA had no effects on the growth of ISV in negative control embryos (Fig. 2A, arrows). Since XDab2 is a short splicing form, we also tested whether the long splicing form of Dab2 could affect ISV formation. Interestingly, overexpression of long splicing form of human Dab2 (p96) or mouse Dab2 (p96) in *Xenopus *embryos also impeded ISV formation (Additional file 1 and 3). The intersomitic veins are formed by sprouting angiogenesis [36]. In *Xenopus*, they appear from stage 30 on by sprouting from the posterior cardinal vein and growing dorsally into the spaces that separate individual somites [37]. Together, these results suggest that both of splicing forms of Dab2 may have conserved functions in ISV formation or angiogenesis.

### XDab2 knockdown leads to defects in blood vessel formation

In order to address whether XDab2 is indispensable for ISV formation, we next carried out the loss-of-function analysis by using antisense morpholino oligonucleotides (MO) capable of depleting XDab2 protein [38]. We designed two morpholino oligonucleotides (MO1 and MO2) which target different regions of *XDab2 *gene to disrupt the translation of *XDab2 *mRNA (Additional file 2. Figure S2A).

To confirm the efficacy and targeting specificity of *XDab2 *MOs, we first coinjected the MOs with C-terminally Myc-tagged *XDab2 *RNAs with or without MO targeting sites in four-cell stage embryos, cultured until stage 12.5, and performed the Western blot analysis with the anti-Myc antibody (Additional file 2. Figure S2B). Coinjection of either MO1 or MO2 inhibited effectively the production of XDab2-Myc protein from RNAs that contain 5' untranslated region (UTR) encoding MO targeting sites but not that from RNAs devoid of 5' UTR. Control MO (Co MO) had no effects on the production of XDab2-Myc, regardless of whether RNAs include MO targeting sites or not. Overall, these indicate the ability of *XDab2 *MO to block specifically the production of *Xenopus *Dab2 protein.

**Figure 3:**
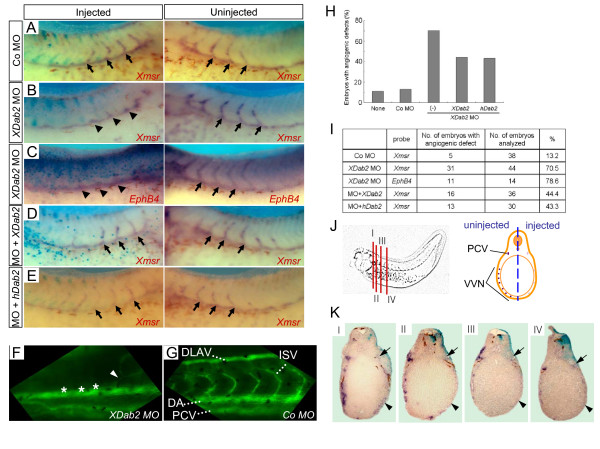
**XDab2 is required for the formation of ISV sprouts**. (A) Control embryos injected with Co MO (30 ng) show no defects in the sprouting ISV. (B-E) *XDab2 *knockdown impedes the formation of ISV and this inhibitory effect can be rescued by coexpression of *Xenopus Dab2 *(D) or human *Dab2 *(E) RNA, which is resistant to the translational inhibition of MO. One blastomere of two-cell stage embryos was injected with *XDab2 *MO (30 ng) alone or with *XDab2 *RNA (250 pg) or *hDab2 *RNA (250 pg), and then embryos fixed at stage 34 were insituhybridized against *Xmsr *(B, D and E) or *EphB4 *(C). Arrows and arrowheads represent the normal and disrupted ISV, respectively. (F and G) Microangiography showing that XDab2 depletion causes abnormality in blood circulation in stage 42 embryos (F: 47%, n = 17), while Co MO-injected embryos reveal the normal circulation (G: 0%, n = 8). Arrowhead and asterisks represent the leaky vessels and the absence of ISV, respectively. DLAV, the dorsal longitudinal anastomosing vessel; DA, the dorsal aorta; PCV, the posterior cardinal veins; ISV, the intersomitic veins. (H and I) The graph and table showing the results from the loss-of-function analysis of *XDab2*. (J and K) Loss-of-function of XDab2 interferes with vasculogenesis. (J) The illustration of transverse section analysis. Roman numerals (I – IV) indicate the positions of embryo sections shown in panel (K). (K) A series of embryo sections show the absence or decrease of the endothelial marker, *Xmsr *in PCV (arrows) and VVN (arrowheads) on the *XDab2 *MO-injected side, which is indicated by the β-galactosidase staining. The *XDab2 *knockdown embryos (n = 12) with the angiogenic defects were analyzed and all of them showed these phenotypes.

We next examined the effects of XDab2 depletion on ISV formation. One blastomere of two-cell stage embryo was injected with *XDab2 *MO (mixture of the same amounts of MO1 and MO2 will be designated *XDab2 *MO hereafter) or Co MO, and the sprouting ISV was observed by hybridizing against endothelial markers at the tadpole stages. As shown in Fig. 3, the sprouting intersomitic veins were absent on the injected side (Fig. 3B and 3C, arrowheads) of *XDab2 *MO-injected embryos as visualized by *Xmsr *or *EphB4 *endothelial markers. And these angiogenic defects could be rescued by coexpression of *XDab2*, human *Dab2 *(p96) or mouse *Dab2 *(p67) mRNA, which cannot bind to MO and is resistant to translation inhibition (Fig. 3D, 3E and Additional file 3). This indicates the specific effects of *XDab2 *MO on the formation of ISV and the functional conservation of splicing forms of Dab2 across species. However, Co MO had no effects on the sprouting ISV (Fig. 3A). Moreover, we performed microangiography to examine the blood circulation in XDab2-depleted embryos at the later stages (Fig. 3F and 3G) [39]. In this experiment, we found that MO-mediated knockdown of *XDab2 *could lead to abnormality in blood supply, causing the leak (Fig. 3F, arrowhead) or absence (Fig. 3F, asterisks) of intersomitic veins while Co MO-injected embryos show normal circulation (Fig. 3G). This indicates that consistent with the angiogenic defects shown by endothelial markers' expression at the earlier stages, XDab2 depletion ultimately can disrupt blood circulation at the advanced stages. Furthermore, we also investigated the effects of XDab2 depletion on the vasculogenic processes including the formation of posterior cardinal vein (PCV) and vitelline vein networks (VVN) (Fig. 3J and 3K). PCV (Fig. 3K, arrows) and VVN (Fig. 3K, arrowheads) in a series of embryo sections were absent or decreased on the *XDab2 *MO-injected side. Taken together, these results indicate that Dab2 is essential for vasculogenesis as well as angiogenesis during *Xenopus *early development.

### Disturbance of activin-like signaling leads to defects in ISV formation

**Figure 4:**
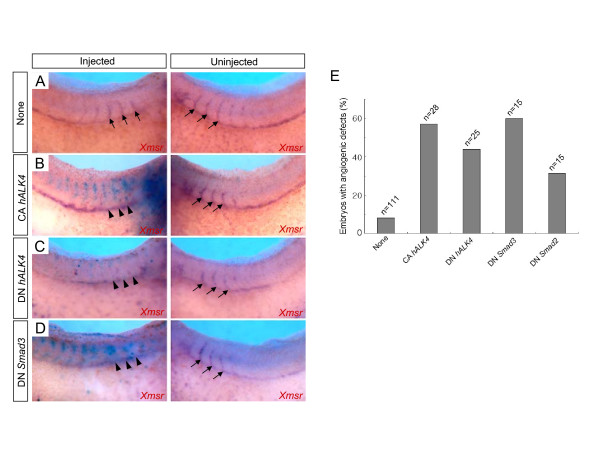
**Gain- and loss-of-function of activin-like signaling inhibit the sprouting of ISV**. One blastomere of two-cell stage embryos was injected with constitutively active activin receptor (CA *hALK4*) DNA, dominant negative activin receptor (DN *hALK4*) DNA or dominant negative *Smad3 *(DN *Smad3*) DNA together with nuclear β-galactosidase mRNA as a lineage tracer and later subjected to insitu hybridization using *Xmsr *probe. (A) Uninjected control embryo. (B) CA *hALK4 *(1 ng)-injected embryo. (C) DN *hALK4 *(1 ng)-injected embryo. (D) DN *Smad3 *(1 ng)-injected embryo. (E) The graph showing the effects of CA *hALK4*, DN *hALK4*, DN *Smad3 *and DN *Smad2 *on the ISV sprouts.

TGFβ is known to induce the expression of *VEGF*, a key molecule in angiogenesis in some cultured cells [20-23]. Thus, we next investigated whether TGFβ or similar signaling pathways would be involved in embryonic angiogenesis during *Xenopus *development. For this purpose, constitutively active activin receptor (CA *hALK4*) [40], dominant negative activin receptor (DN *hALK4*) [41], dominant negative *Smad2 *(DN *Smad2*) or dominant negative *Smad3 *(DN *Smad3*) [42] DNA was injected into one blastomere of two-cell stage embryos and then their effects on ISV formation was examined by in situ hybridization analysis using *Xmsr *probe (Fig. 4). We excluded embryos exhibiting abnormal morphology which is probably due to the effect of other functions of activin-like signaling pathway during embryogenesis but analyzed normal-looking embryos at early tadpole stages. In these analyses, injection of CA *hALK4 *or DN *hALK4 *caused defects in ISV formation on the injected side of embryos to a similar degree (Fig. 4B and 4C). In addition, functional inhibition of Smad3, a downstream effector of TGFβ signaling, by expression of DN *Smad3 *disrupted the sprouting of ISV (Fig. 4D). DN Smad2 had also inhibitory effects on ISV formation, but to a lesser extent than DN Smad3. Overall, these data suggest that activin-like signaling may affect on normal embryonic angiogenesis during *Xenopus *early development.

### Activin-like signaling regulates ISV formation through XDab2-mediated VEGF induction

**Figure 5:**
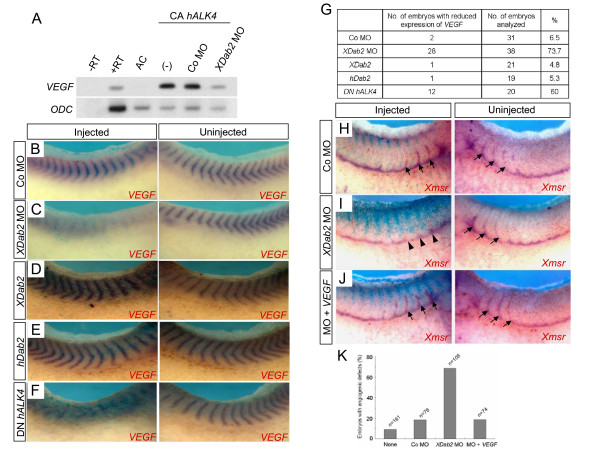
**XDab2 mediates the induction of *VEGF *by activin-like signaling**. (A) RT-PCR analysis revealing that *XDab2 *MO (40 ng), but not Co MO, inhibits the induction of *VEGF *gene by CA *hALK4 *RNA (2 ng) in animal cap tissues. +RT and -RT; control RT-PCR on the whole embryo RNA in the presence or absence of reverse transcriptase. AC, uninjected animal cap cells. Four-cell stage embryos were injected into the animal pole region with a combination of the indicated reagents, and then animal caps isolated at late blastula stages were cultured to stage 20 and subsequently subjected to RT-PCR analysis. (B-E) *VEGF *gene expression could be reduced by Dab2 depletion but not by its overexpression. (D) *XDab2 *RNA (2 ng)-injected embryo. (E) *hDab2 *RNA (2 ng)-injected embryo. (F) The inhibition of activin-like signaling by injection of DN *hALK4 *(1 ng) decreased *VEGF *gene expression. (G) The table summarizing the results of Fig. 5B-F. (H-J) The angiogenic defects caused by *XDab2 *knockdown can be rescued by coexpression of *VEGF*. Arrows and arrowheads indicate the normal and inhibited ISV, respectively. The amount of injected reagents: 30 ng, Co Mo; 30 ng, *XDab2 *MO; 1 ng, *VEGF *mRNA. (K) The graph showing the results of Fig. 5H-J.

Our results revealed that XDab2 and activin-like signal are essential for ISV formation in developing early embryos. Since Dab2 acts as an adaptor to mediate TGFβ signaling pathway [32], we next examined whether XDab2 might function downstream of activin-like signaling pathway to mediate *VEGF *gene expression for the regulation of blood vessel formation. To this end, we first performed RT-PCR analysis to test whether XDab2 mediates the induction of *VEGF *by activin-like signal (Fig. 5A). Functional analysis in *Xenopus *animal caps showed that injection of constitutively active activin receptor (CA *hALK4*) could induce *VEGF *gene, and this induction could be interfered by coexpression of *XDab2 *MO. Co MO, however, did not affect the expression of *VEGF*. We also carried out this analysis using *Xnr1 *(*Xenopus *nodal-related 1), a ligand of Nodal signaling, with the same result (data not shown). Together, these results suggest that XDab2 may mediate the induction of *VEGF *by activin-like signaling. Furthermore, we examined whether XDab2 depletion also inhibit the expression of endogenous *VEGF *in whole embryos. For this purpose, we injected Co MO or *XDab2 *MO into one blastomere of two-cell stage embryos and then performed *in situ *hybridization analysis using *VEGF *probe [24] at the embryonic stages just prior to the formation of blood vessels including PCV and ISV (Fig. 5B and 5C). *VEGF *gene expression was decreased markedly on the injected side of *XDab2 *MO-injected embryos (Fig. 5C) whereas it was unchanged in the Co MO-injected embryos (Fig. 5B). Since overexpression of *Dab2 *or DN *hALK4 *could inhibit ISV formation as shown above, we also examined their effects on the expression of endogenous *VEGF*. As shown in Fig. 5D and 5E, injection of *XDab2 *or *hDab2 *did not induce significant changes in VEGF expression, though a small percentage of injected embryos exhibited its increased pattern (data not shown). In contrast, DN *hALK4*-mediated inhibition of activin-like signaling suppressed *VEGF *expression (Fig. 5F). Overall, these data suggest that Dab2-mediated activin-like signaling is essential for *VEGF *expression in vivo during *Xenopus *development.

As VEGF has a key role in angiogenesis, we next asked whether the XDab2-mediated induction of *VEGF *is relevant to ISV formation in *Xenopus *early embryos. To test this, we examined whether the angiogenic defects caused by XDab2 depletion could be rescued by coexpression of *VEGF *(Fig. 5H–J). In this experiment, injection of *XDab2 *MO inhibited the formation of intersomitic veins as described above (Fig. 5I, arrowheads), and this inhibition could be rescued by coinjection of *Xenopus VEGF *mRNA [24,25] (Fig. 5J). These data indicate that the XDab2-mediated induction of *VEGF *may regulate the growth of intersomitic veins in early embryos.

**Figure 6:**
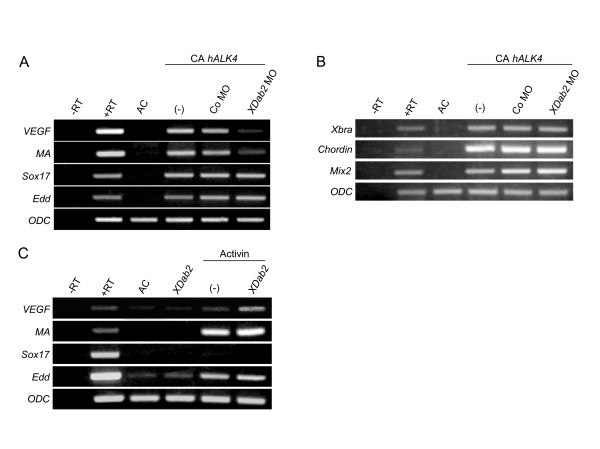
**XDab2 acts as a specific mediator of activin-like signaling for *VEGF *gene induction**. (A and B) Depletion of XDab2 inhibits the induction by activin-like signal of late mesodermal target genes such as *VEGF *and muscle actin (*MA*) without affecting that of early mesodermal (*Chordin*, *Xbra *and *Mix2*) and late endodermal (*Sox17 *and *Endodermin*) target genes in animal caps. (C) XDab2 enhances the activity of activin protein to induce *VEGF*, but the expression of other target genes was not changed by *XDab2 *overexpression. (A-C) Four-cell stage embryos were injected into the animal pole region with a combination of the indicated reagents and the animal caps isolated at stage 8 were cultured to stage 10.5 (B) or 20 (A and C) and then subjected to RT-PCR analysis. The amount of the injected reagents: 2 ng, CA *hALK4 *RNA; 40 ng, Co MO; 40 ng, *XDab2 *MO; 2 ng, *XDab2 *RNA. In panel (C), the animal caps were cultured in the presence of 5 ng/ml of activin protein. +RT and -RT; control RT-PCR on the whole embryo RNA in the presence or absence of reverse transcriptase. AC, uninjected animal cap cells.

Activin-like signals have a variety of roles during the patterning of *Xenopus *early embryos, and many other target genes as well as *VEGF *are induced by these signals in animal caps. We thus tested whether XDab2 also mediates the induction of other target genes by activin-like signals (Fig. 6). Intriguingly, our results showed that *XDab2 *MO inhibited the induction of late mesodermal markers including *VEGF *and muscle actin (*MA*), but not that of endodermal markers including endodermin (*Edd*) or *Sox17 *genes by expression of CA *hALK4 *in animal caps (Fig. 6A). In addition, XDab2 depletion did not affect the induction of earlier mesodermal markers such as *Xbra*, *Chordin *and *Mix2 *by activin-like signals (Fig. 6B). Consistently, XDab2 could enhance the activity of activin ligand in the induction of *VEGF *gene only, but not in that of other target genes (Fig. 6C). These results suggest that XDab2 may function as a specific adaptor to induce *VEGF *downstream of activin-like signaling pathway.

## Discussion

In this study, we have demonstrated that activin-like signaling pathway is implicated in angiogenesis in *Xenopus *early embryos and Dab2 acts as a specific mediator for this process. First, *Xenopus Dab2 *is specifically expressed in vasculatures of early embryos including intersomitic veins (ISV). Second, overexpression and depletion of XDab2 interferes with the sprouting of ISV. In addition, the up- and down-regulation of activin-like signaling by expression of CA *hALK4*, DN *hALK4*, DN *Smad2 *or DN *Smad3 *affects the formation of ISV. Finally, XDab2 is required for the induction by activin-like signals of *VEGF*, a critical angiogenic factor, with no effects on that of other target genes. Together, these results suggest the possible signaling cascade that regulates the early vascular development in vertebrates.

### Angiogenic defects caused by the gain- and loss-of-function of XDab2

Our results show that both overexpression and knockdown of *XDab2 *can cause the disappearance of the intersomitic veins (Figs. 2 and 3). This angiogenic defect by XDab2 depletion may be due to the loss of XDab2-mediated *VEGF *expression. Consistent with this, analysis of heterozygous *VEGF *mutant embryos, which are less affected than those homozygous for this mutation, revealed a strong decrease in ISV sprouts [7]. Moreover, even loss of a single *VEGF *allele results in embryonic lethality at E11.5, indicating a strict dose-dependent regulation of embryonic blood vessel development by VEGF [7,10,43]. Then, the next question to address is how the absence of the sprouting ISV could be caused by *XDab2 *overexpression, although several molecules involved in embryonic angiogenesis show similar phenotypes in the gain-and loss-of-function analysis [37,44]. Two possibilities could account for this finding. First, VEGF increased by XDab2 may interfere with the formation of angiogenic vessels. In quail embryogenesis, the intersomitic arteries in the *VEGF*-injected halves were either missing or stunted, whereas in the uninjected halves, the intersomitic and vertebral vessel development was normal [45]. This indicates that ectopic expression of *VEGF *could induce angiogenic defects. In addition, loss of *ALK1*, which is a member of TGFβ type I receptors and activates Smad1/5/8 effectors, leads to up-regulation of angiogenic factors such as VEGF and Ang-2 (angiopoietin-2). Nevertheless, *ALK1 *knockout mice exhibited defective angiogenesis and vascular smooth muscle cell development, although endothelial differentiation and vasculogenesis appear normal [46]. Second, it is possible that up-regulation of activin-like signaling by overexpression of *XDab2 *may induce anti-angiogenic factors as well as *VEGF*. TGFβ can both stimulate and inhibit proliferation of endothelial cells. Low doses of TGFβ stimulate proliferation and migration of endothelial cells, while high doses of TGFβ inhibit these responses [47]. Recent evidence has shown that TGFβ signaling could induce both the angiogenic molecule, *VEGF*, and the anti-angiogenic molecules such as thrombospondin-1 (TSP-1), known as a major antiangiogenic factor, and soluble Flt-1 (sFlt-1), which is a soluble receptor and antagonist of VEGF [48]. In light of this, it is tempting to speculate that XDab2-promoted activin-like signaling may stimulate the expression of anti-angiogenic factors in *Xenopus *early embryos, and then these factors could inhibit the function of VEGF. The role of anti-angiogenic factors including TSP-1 and sFlt-1 and their relationship with activin-like signaling in angiogenesis during *Xenopus *early development remain to be investigated.

### Dab2-mediated activin-like signaling in angiogenesis

Our gain- and loss-of-function analysis of activin-like signaling suggests its possible role in angiogenesis at later stages of *Xenopus *early development. Consistently, depletion of TGFβ and its receptors has demonstrated the critical role of TGFβ signaling in vascular development. TGFβ1-deficient mice die in utero due to vascular defects [49] and loss of TGFβ type I or type II receptor in mice results in embryonic lethality at around E10.5 due to defects in vascular development of the yolk sac [50,51]. In addition, several reports have shown that TGFβ signaling induces *VEGF *gene expression in cultured cells [18-23]. However, the molecular mechanisms underlying the regulation of *VEGF *gene expression in whole organisms are poorly understood.

Dab2 is known to function as a component of TGFβ signaling by linking TGFβ receptors and Smad proteins in cultured cells [32]. This suggests the possibility that XDab2 regulates embryonic angiogenesis through activin-like signaling pathway. Supporting this hypothesis, unlike wild-type XDab2, its mutant devoid of PTB domain, which mediates its interaction with TGFβ receptors and Smad2/3, could not inhibit the sprouting of ISV when overexpressed in early embryos (data not shown). Moreover, XDab2 is not only required for but also capable of augmenting the ability of activin signal to induce *VEGF *gene (Fig. 6C). These results indicate that XDab2 lies in the activin-like signaling cascade for the regulation of the angiogenic events. Interestingly, our results reveal that depletion of XDab2 affect the induction by activin signals of *VEGF *and *MA *genes, but not that of other early and late target genes (Fig. 6). Given the expression of *XDab2 *in the somites and vascular structures of early embryo, this indicates that Dab2-mediated activin-like signaling may be involved in somite tissue specification as well as angiogenesis. However, since activin-like signaling could affect the late mesoderm specification in the somites that is critical for blood vessel formation, we cannot exclude the possibility that it could have indirect effects on vascular development. Nevertheless, it is worth noting that activin-like signaling employs specific mediators such as Dab2 only for the late specific developmental events but not for the early ones in *Xenopus *embryos. In line with this, the gain- and loss-of-function of XDab2 had no effects on the axis formation and patterning of early embryos that are regulated by Wnt and activin-like signaling pathway (data not shown), though it is also known as an inhibitor of Wnt signaling [33], indicating its specific function in late mesoderm specification and angiogenesis as shown our data in *Xenopus *development. Although it is known that other adaptor proteins such as SARA [52], Dok-1 [53], Axin [54], the ELF β-spectrin [55] and cPML [56] are involved in TGFβ signaling pathway, it remains to be further investigated whether these adaptors also function in angiogenesis or other specific events regulated by TGFβ signaling in whole organisms.

### Functional conservation of Dab2 in blood vessel development

During mouse embryogenesis, mouse *Dab2 *(*mDab2*) expression is first observed in the primitive endoderm at E4.5 and it is still restricted to the visceral endoderm at E7.5 [29,30]. The homozygous *Dab2*-deficient mutant is embryonic lethal (earlier than E6.5) due to the defective visceral endoderm formation [29]. The conditional null mice for *Dab2 *show defects in kidney function such as reduction of transport by megalin, a lipoprotein receptor, in the proximal tubule, but the kidney appeared grossly normal, despite the absence of Dab2 protein that is normally expressed in the kidney proximal tubule cells [30]. *Dab2 *is also highly expressed in a variety of adult tissues, including the kidney, ovary, liver, mammary gland, intestine, uterus and heart [57]. *Dab2 *conditionally null mice also appeared normal when *Dab2*-expressing organs such as kidney, intestine and brain were analyzed [30]. However, there is no report that mDab2 is involved in angiogenesis during mouse embryogenesis yet. Although these embryonic roles of mDab2 in mouse embryogenesis seem to differ from those of XDab2 in many respects, we think that the function of mDab2 in angiogenesis needs to be investigated in the future research. On the other hand, a recent study reported that zebrafish *Dab2 *(*zDab2*) is expressed in caudal vein (CV) and dorsal aorta (DA) [58], indicating its possible roles in blood vessel development. Furthermore, we showed that the angiogenic defects by *XDab2 *knockdown could be rescued by either human or mouse *Dab2 *genes (Fig. 3E and Additional file 3). Together, these results suggest that the angiogenic function of Dab2 may be conserved during vertebrate development.

Dab2 has two splicing variants including long (p96) and short (p67) forms in mammals. Compared with the long variant in mice, the short one lacks the exon 8 containing two DPF and two of five NPF motifs which are implicated in endocytosis. While p96 isoform is essential for normal endocytosis and mouse development, expression of p67 alone led to decreased endocytosis and delayed development [59], suggesting their distinct functions. Nevertheless, our study reveals that both of splicing isoforms have similar functions in blood vessel development.

Supporting this, both of long and short splicing isoforms of Dab2 disrupted similarly the sprouting of intersomitic veins when overexpressed. Moreover, the angiogenic defects caused by XDab2 depletion could be recovered by coinjection of either. Thus, it is tempting to speculate that the motifs critical for endocytosis which the long splicing isoform has might be dispensable for blood vessel formation. Possibly, Dab2 could function as a signaling mediator but not as an adaptor for endocytosis in the angiogenic events. On the other hand, it is possible that overexpression of short splicing isoforms such as XDab2 can inhibit the ISV formation by competing off the long isoforms which might be more relevant to blood vessel formation. However, given that overexpression of *XDab2 *cannot reduce *VEGF *expression that is critical for angiogenesis (Fig. 5), its inhibitory effects on the ISV formation might not be due to the impediment of the long isoform's function. Like *XDab2*, zebrafish *Dab2 *expressed in blood vessels is more similar to short isoforms than long ones (Tab.1). Probably, in lower vertebrates such as fish and frog, a short isoform of Dab2 alone may play the same roles that its two isoforms have in human and mice during blood vessel development. In the future, it will be necessary to elucidate the molecular mechanism by which Dab2 regulates *VEGF *expression in activin-like signaling pathways.

## Conclusion

In summary, our study shows that Dab2 has a pivotal role in embryonic angiogenesis during *Xenopus *early development. In this process, it functions as a specific mediator of *VEGF *induction by activin-like signaling pathway. The detailed mechanism governing the function of Dab2 in vasculature, its significance in pathological angiogenesis, and its functional conservation in other vertebrate development remain to be elucidated.

## Methods

### Xenopus embryos and microinjection

*Xenopus laevis *was purchased from Xenopus I (Ann Arbor, MI). Eggs were obtained from *Xenopus laevis *primed with 800 units of human chorionic gonadotropin (Sigma). In vitro fertilization was performed as described previously [60], and developmental stages of the embryos were determined according to Nieuwkoop and Faber [61]. Microinjection was carried out in 0.33 × Modified Ringer (MR) containing 4% Ficoll-400 (Sigma) using a Nanoliter Injector (WPI). Injected embryos were cultured in 0.33 × MR until stage 8 and then transferred to 0.1 × MR until they had reached the appropriate stage for the experimentation outlined below.

### Plasmids, RNA synthesis, and morpholino oligonucleotides

For expression in *Xenopus *embryos, the entire coding region of *XDab2 *was cloned into the *ClaI *and *XbaI *sites of the pCS2+ vector and into the *ClaI *site of the Myc-pCS2+ vector. Capped mRNAs were synthesized from linearized plasmids using the mMessagae mMachine kit (Ambion). *XDab2 *and *XDab2*-Myc were linearized with *NotI*, and mRNA was synthesized using SP6 RNA polymerase. Antisense morpholino oligonucleotides (MO) were obtained from Gene Tools. The morpholino oligonucleotide sequences were as follows: *XDab2 *MO1, 5'-CTACATCAGTAGACATGACTGGAGG-3'; *XDab2 *MO2, 5'-CACAATCATTAAATAAGAG TCAGAT-3'; control MO, 5'-CCTCTTACCTCAGTTACAATTTATA-3'. DN *hALK4 *and CA *hALK4 *in the pCS2+ vector were linearized with *NotI*. DN *Smad2 *in the pSP64T vector was linearized with *XbaI*. pCS2-DN *Smad3*, pCS2-mDab2 (p96), pCS2-mDab2 (p67), pCS2-hDab2 and pCS2-xVEGF were generated by subcloning of the coding regions of pRK5-DN *Smad3*, pGEX-KG-mDab2 (p96), pGEX-KG-mDab2 (p67), pBSK-xVEGF and pRK5-hDab2. These were linearized with *NotI *for in vitro RNA synthesis.

### In situ hybridization and sectioning of embryos

Whole-mount in situ hybridization was performed with digoxigenin (DIG)-labeled probes as described by Harland [62]. Anti-sense insitu probes against *XDab2 *and *Xmsr *were generated by linearizing the pBSKII-*XDab2 *construct with *BamHI *and pGEM7zf-*Xmsr *construct with *EcoRI*, respectively and transcribing with SP6 RNA polymerase. *EphB4 *and *VEGF *RNA probes are described in [24,37].

For sectioning, embryos were fixed in MEMFA and then rinsed three times in phosphate-buffered saline (PBS) and soaked for 1 hour in 0.4% sucrose in PBS. Subsequently, the embryos were washed in PBS and embedded in 4% low-melting temperature agarose (Sigma). Embedded embryos were sectioned per 50 μm on a Vibratome Series 1000 Plus (Vibratome).

### Microangiography

We anesthetized stage 42 tadpoles in 0.02% ethyl 3-aminobenzoate methanesulfonate (MS222, Sigma) dissolved in 0.1× MR, placed them onto the agarose coated dish and injected FITC-dextran (Sigma) into their hearts using glass needles placed on a micromanipulator. We used fluorescent microscopy (Axiovert 200 M, Carl Zeiss) for imaging.

### RT-PCR

Total RNA was prepared from embryos or animal cap explants with TRI reagent (Sigma) and treated with RNase-free DNase I (Roche) to remove genomic DNA. RNA was transcribed by using M-MLV reverse transcriptase (Promega). PCR amplification was performed using *Taq *polymerase (TaKaRa). Primers and amplification cycles for RT-PCR analysis were as follows:

*XDab2 *forward, 5'-CACTGGAAGCCTTGGCACCT-3'; *XDab2 *reverse, 5'-CCTTGTTGC GGCCAAACATT-3' (25 cycles); *VEGF *forward, 5'-TACATCCCCCATGCCCAGTT-3'; *VEGF *reverse, 5'-TCTCATCAGGG GCACACAGG-3' (25 cycles); Primers for *ODC*, *Sox17*, *Edd*, *MA*, *Xbra*, *Chordin *and *Mix2 *were as described in Dr. De Robertis' homepage [63].

### Western blotting analysis

For Western Blot analysis to test *XDab2 *MO specificity, 5'UTR and ORF *XDab2*-Myc mRNA were injected with *XDab2 *MO1, MO2 or control MO into the animal region of embryos at the four-cell stage and then the embryos cultured until early neurula stage were homogenized in Triton X-100 lysis buffer (20 mM Tris, 1% Triton X-100, 140 mM NaCl, 10% glycerol, 1 mM EGTA, 1.5 mM MgCl2, 1 mM DTT, 1 mM sodium vanadate, 50 mM NaF, 10 μg/ml aprotinin, 10 μg/ml leupeptin). Equal amounts of protein were separated by 10% SDS-PAGE. Western blots were performed according to standard protocol with anti-Myc (1:1000, Santa Cruz) and anti-actin (1:1000, Santa Cruz) antibodies. Actin served as a specificity control.

## Authors' contributions

SMC carried out all experiments, participated in its design and drafted the manuscript. SCC participated in the design of study and coordination and helped to draft the manuscript. JKH conceived of study and participated in its design and coordination and helped to draft the manuscript. All authors read and approved the final manuscript.

## Supplementary Material

Additional file 1 **Gain-of-function of *hDab2 *impedes the sprouting of ISV in *Xenopus *embryo**. (A) Injection of *hDab2 *RNA (2 ng) inhibited the formation of the sprouting ISV on the injected side of the embryo, and that on the uninjected side was normal. One blastomere of two-cell stage embryos was injected with *hDab2 *RNA along with β-gal RNA as a lineage tracer. Embryos were fixed at stage 34, stained for β-gal and then hybridized against *Xmsr *or *EphB4*. Arrowheads indicate disrupted ISV on the injected side of the embryo. Rectangular areas in the upper panels are enlarged in the lower panels. (B) The table summarizing the results from the gain-of-function analysis of *hDab2*. Click here for file

Additional file 3 **Splicing isoforms of *mDab2 *have similar effects on the ISV formation in *Xenopus *embryo**. (A) Injection of Co MO (30 ng) caused no defects in the sprouting ISV. (B-D) *XDab2 *knockdown inhibited the formation of ISV (B) and this angiogenic defect could be rescued by coexpression of *mDab2 p67 *(C) or *mDab2 p96 *(D) RNA. One blastomere of two-cell stage embryos was injected with *XDab2 *MO (30 ng) with or without *mDab2 p67 *or *p96 *RNA (250 pg), and then embryos fixed at stage 34 were in situhybridized against *Xmsr*. Arrows and arrowheads represent the normal and disrupted ISV, respectively. (E) The table showing the results of Figure S3A-D. (F-I) Gain-of-function of mDab2 p67 or p96 also disrupts the sprouting of ISV in *Xenopus *embryo. Injection of *mDab2 p67 *(F and G) or *p96 *(H and I) RNA (2 ng) inhibits the formation of the sprouting ISV on the injected side of the embryo, with that on the uninjected side being normal. One blastomere of two-cell stage embryos was injected with β-gal RNA as a lineage tracer with *mDab2 p67 *or *p96 *RNA. Embryos were fixed at stage 34, stained for β-gal and then hybridized against *Xmsr *(F and H) or *EphB4 *(G and I). Arrows and Arrowheads indicate normal and disrupted ISV on the injected side of the embryo, respectively. (J) The table showing the results of Figure S3F-I. Click here for file

Additional file 2 **The efficacy and targeting specificity of *XDab2 *MO**. (A) The diagram indicating MO targeting site. (B) *XDab2 *MO inhibits specifically the translation of its cognate mRNA, but Co MO cannot. C-terminally Myc-tagged *XDab2 *mRNA (1 ng) with or without MO targeting site was coinjected with Co MO (40 ng), MO1 (40 ng) or MO2 (40 ng) into the four-cell stage embryos, and then embryos sampled at the early gastrula stages were subjected to western blotting analysis. Actin serves as a loading control. 5'UTR, *XDab2*-Myc mRNA with MO targeting site; ORF, *XDab2*-Myc mRNA without MO targeting site. Click here for file
