# Supplementary material for: Xenopus Dab2 is required for embryonic angiogenesis
Source: BMC Dev Biol. 2006 Dec 19;6:63. doi: 10.1186/1471-213X-6-63 (PMC1766927; doi:10.1186/1471-213X-6-63)
Supplement: Additional file 1 — Gain-of-function of hDab2 impedes the sprouting of ISV in Xenopus embryo. (A) Injection of hDab2 RNA (2 ng) inhibited the formation of the sprouting ISV on the injected side of the embryo, and that on the uninjected side was normal. One blastomere of two-cell stage embryos was injected with hDab2 RNA along with β-gal RNA as a lineage tracer. Embryos were fixed at stage 34, stained for β-gal and then hybridized against Xmsr or EphB4. Arrowheads indicate disrupted ISV on the injected side of the embryo. Rectangular areas in the upper panels are enlarged in the lower panels. (B) The table summarizing the results from the gain-of-function analysis of hDab2. [file 1471-213X-6-63-S1.pdf]

A

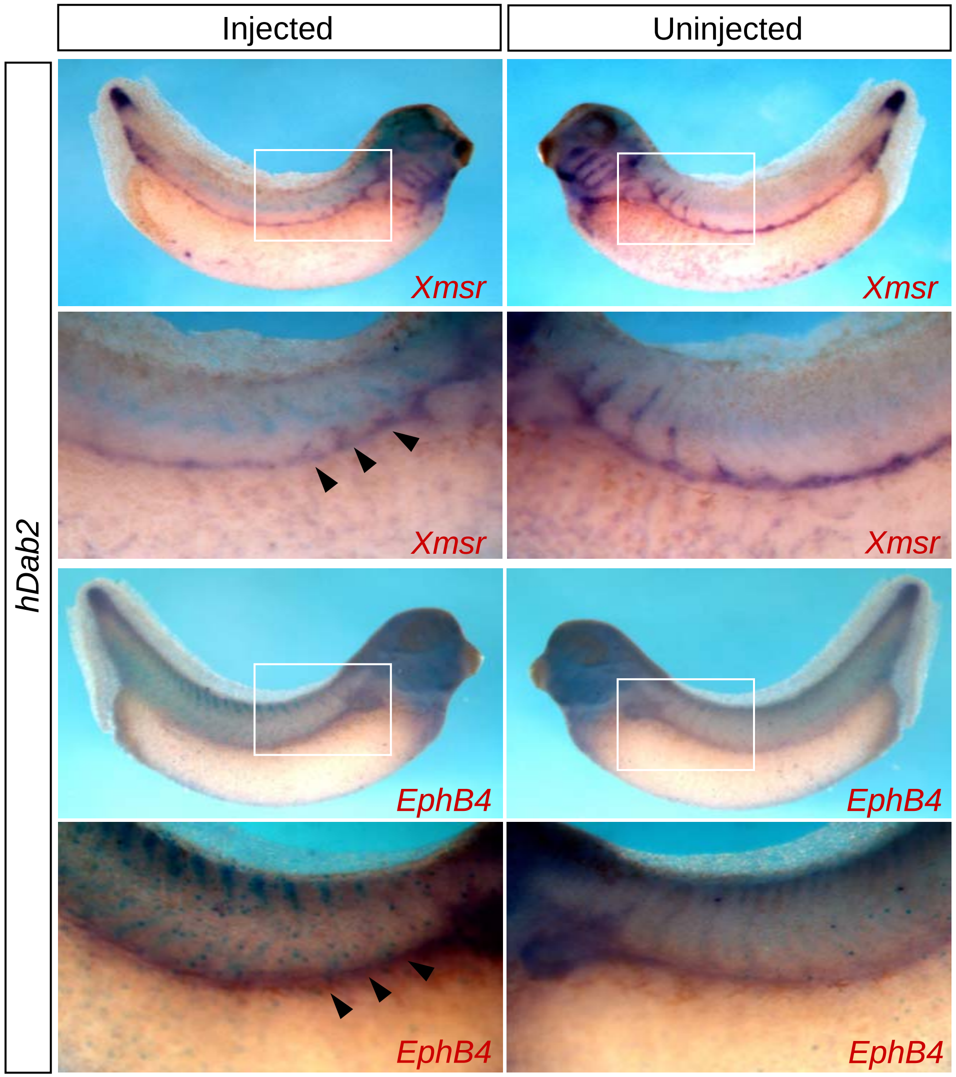

B

probe	RNA	No. of embryos with angiogenic defect	No. of embryos analyzed	%
Xmsr	*β-gal*	1	13	7.7
	hDab2	13	32	40.6
EphB4	*β-gal*	2	19	10.5
	hDab2	9	19	47.4
