# Supplementary material for: Xenopus Dab2 is required for embryonic angiogenesis
Source: BMC Dev Biol. 2006 Dec 19;6:63. doi: 10.1186/1471-213X-6-63 (PMC1766927; doi:10.1186/1471-213X-6-63)
Supplement: Additional file 3 — Splicing isoforms of mDab2 have similar effects on the ISV formation in Xenopus embryo. (A) Injection of Co MO (30 ng) caused no defects in the sprouting ISV. (B-D) XDab2 knockdown inhibited the formation of ISV (B) and this angiogenic defect could be rescued by coexpression of mDab2 p67 (C) or mDab2 p96 (D) RNA. One blastomere of two-cell stage embryos was injected with XDab2 MO (30 ng) with or without mDab2 p67 or p96 RNA (250 pg), and then embryos fixed at stage 34 were in situhybridized against Xmsr. Arrows and arrowheads represent the normal and disrupted ISV, respectively. (E) The table showing the results of Figure S3A-D. (F-I) Gain-of-function of mDab2 p67 or p96 also disrupts the sprouting of ISV in Xenopus embryo. Injection of mDab2 p67 (F and G) or p96 (H and I) RNA (2 ng) inhibits the formation of the sprouting ISV on the injected side of the embryo, with that on the uninjected side being normal. One blastomere of two-cell stage embryos was injected with β-gal RNA as a lineage tracer with mDab2 p67 or p96 RNA. Embryos were fixed at stage 34, stained for β-gal and then hybridized against Xmsr (F and H) or EphB4 (G and I). Arrows and Arrowheads indicate normal and disrupted ISV on the injected side of the embryo, respectively. (J) The table showing the results of Figure S3F-I. [file 1471-213X-6-63-S3.pdf]

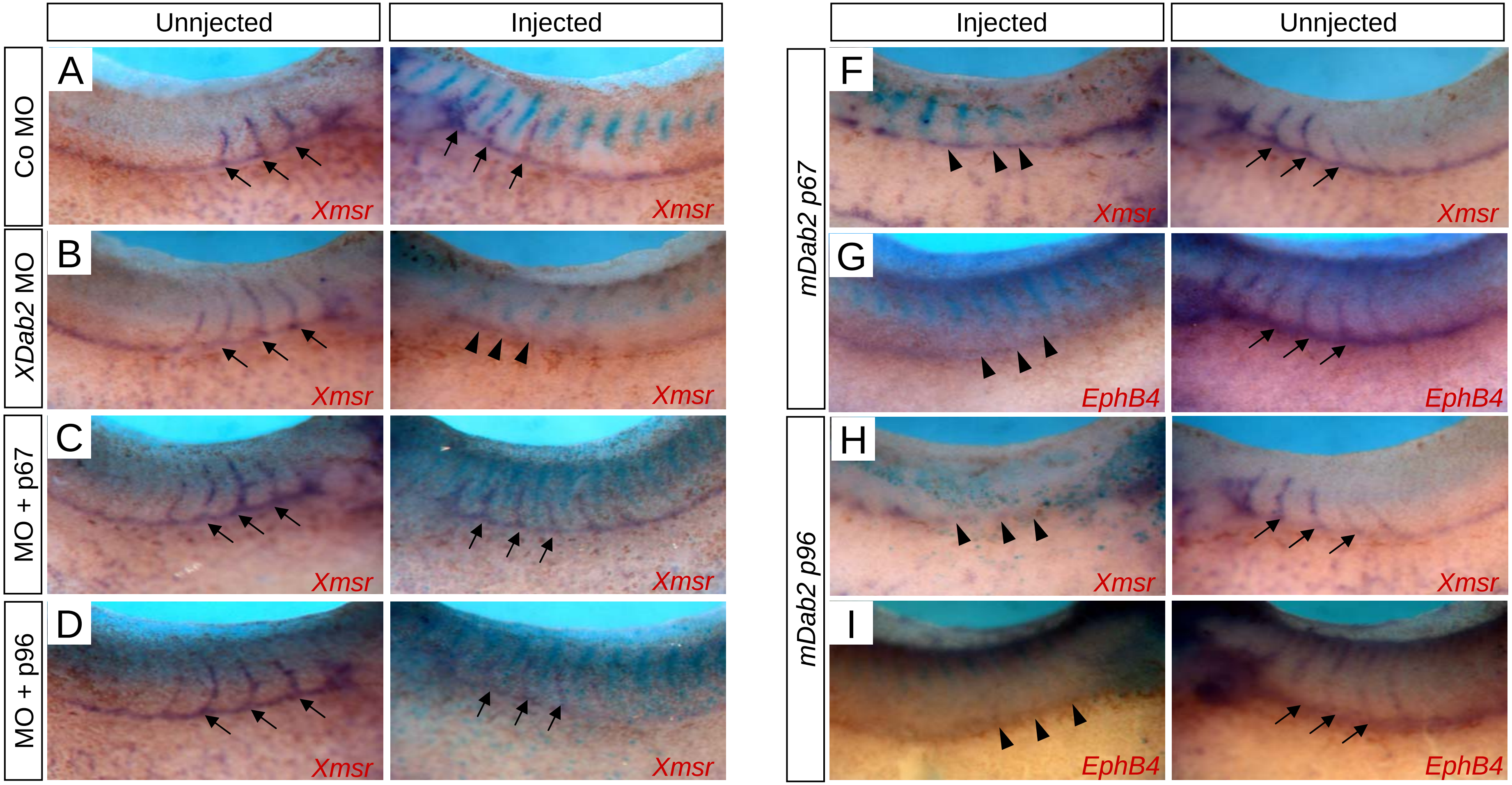

E

	No. of embryos with angiogenic defect	No. of embryos analyzed	%
Co MO	2	35	5.7
XDab2 MO	19	26	73.1
MO+m*Dab2 p67*	10	24	41.7
MO+*mDab2 p96*	13	29	44.8

J

probe	RNA	No. of embryos with angiogenic defect	No. of embryos analyzed	%
Xmsr	*β-gal*	2	37	5.4
	mDab2 p67	8	15	53.3
	mDab2 p96	10	21	47.6
EphB4	*β-gal*	2	28	7.1
	mDab2 p67	9	17	52.9
	mDab2 p96	12	23	52.2
