# Supplementary figures and images for: Xenopus Dab2 is required for embryonic angiogenesis
Source: BMC Dev Biol. 2006 Dec 19;6:63. doi: 10.1186/1471-213X-6-63 (PMC1766927; doi:10.1186/1471-213X-6-63)

**A**

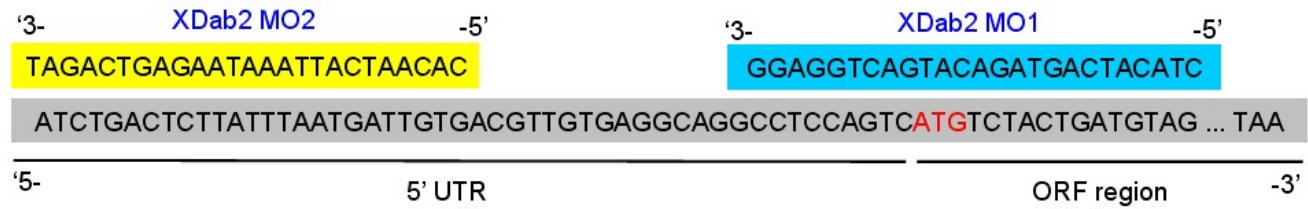

B

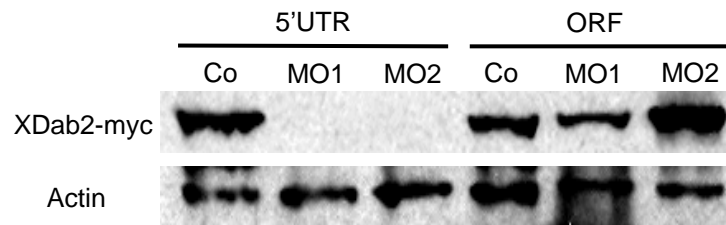

Supplement: Additional file 2 — The efficacy and targeting specificity of XDab2 MO. (A) The diagram indicating MO targeting site. (B) XDab2 MO inhibits specifically the translation of its cognate mRNA, but Co MO cannot. C-terminally Myc-tagged XDab2 mRNA (1 ng) with or without MO targeting site was coinjected with Co MO (40 ng), MO1 (40 ng) or MO2 (40 ng) into the four-cell stage embryos, and then embryos sampled at the early gastrula stages were subjected to western blotting analysis. Actin serves as a loading control. 5'UTR, XDab2-Myc mRNA with MO targeting site; ORF, XDab2-Myc mRNA without MO targeting site. [file 1471-213X-6-63-S2.pdf]
